# Quantification of early learning and movement sub-structure predictive of motor performance

**DOI:** 10.1038/s41598-021-93944-9

**Published:** 2021-07-13

**Authors:** Vikram Jakkamsetti, William Scudder, Gauri Kathote, Qian Ma, Gustavo Angulo, Aksharkumar Dobariya, Roger N. Rosenberg, Bruce Beutler, Juan M. Pascual

**Affiliations:** 1 Rare Brain Disorders Program, Department of Neurology, The University of Texas Southwestern Medical Center, 5323 Harry Hines Blvd., Mail Code 8813, Dallas, TX 75390-8813 USA; 2 Center for the Genetics of Host Defense, The University of Texas Southwestern Medical Center, Dallas, TX USA; 3 Department of Physiology, The University of Texas Southwestern Medical Center, Dallas, TX USA; 4 Department of Pediatrics, The University of Texas Southwestern Medical Center, Dallas, TX USA; 5 Eugene McDermott Center for Human Growth and Development/Center for Human Genetics, The University of Texas Southwestern Medical Center, Dallas, TX USA

**Keywords:** Neuroscience, Motor control, Social behaviour

## Abstract

Time-to-fall off an accelerating rotating rod (rotarod) is widely utilized to evaluate rodent motor performance. We reasoned that this simple outcome could be refined with additional measures explicit in the task (however inconspicuously) to examine what we call movement sub-structure. Our goal was to characterize normal variation or motor impairment more robustly than by using time-to-fall. We also hypothesized that measures (or features) early in the sub-structure could anticipate the learning expected of a mouse undergoing serial trials. Using normal untreated and baclofen-treated movement-impaired mice, we defined these features and automated their analysis using paw video-tracking in three consecutive trials, including paw location, speed, acceleration, variance and approximate entropy. Spectral arc length yielded speed and acceleration uniformity. We found that, in normal mice, paw movement smoothness inversely correlated with rotarod time-to-fall for the three trials. Greater approximate entropy in vertical movements, and opposite changes in horizontal movements, correlated with greater first-trial time-to-fall. First-trial horizontal approximate entropy in the first few seconds predicted subsequent time-to-fall. This allowed for the separation, after only one rotarod trial, of different-weight, untreated mouse groups, and for the detection of mice otherwise unimpaired after baclofen, which displayed a time-to-fall similar to control. A machine-learning support vector machine classifier corroborated these findings. In conclusion, time-to-fall off a rotarod correlated well with several measures, including some obtained during the first few seconds of a trial, and some responsive to learning over the first two trials, allowing for predictions or preemptive experimental manipulations before learning completion.

## Introduction

The rotating rod, or rotarod, performance test is a standard assay of motor behavior in rodents widely utilized in the assessment of both physiological variants and disease models. This is due, at least in part, to the scalability of the task from single mutant mice to the broader-scale screening of hundreds of mice^1–6^. The assay most commonly comprises a number of trials wherein a rodent is placed on a rotating accelerating rod and its ability to stay balanced on it is reported as ‘time-to-fall’^7^. With each trial, normal rodents learn to adjust their paw position patterns to adapt to the accelerating contact surface^4^. At the end of a trial, the total duration of a mouse on the rod (time-to-fall) is broadly utilized as a quantitative indicator of the motor ability of the mouse. Using this method, mouse performance normally increases sharply with each of the first few trials and then, more slowly, reaches a maximum or plateau, similarly to numerous other forms of learning based on practice or repetition. Nevertheless, only the time-to-fall (also called rotarod score) after reaching the maximal, plateau stage is favored over other aspects or features of motor learning when comparing motor abilities across groups of mice. In fact, the information acquired from the first few trials of a mouse is often limited to the success of the habituation—or lack thereof—of the mouse to the apparatus. However, the first exposures of a mouse to a new task can also provide information related to its spontaneous motor proclivity before it changes with motor learning. Moreover, the most common measure derived from the rotarod performance test is a single number at the end of the session; i.e., either the total duration spent on the rotarod, or the revolutions per minute (RPM or angular velocity) of the rotarod when the mouse falls. Furthermore, rotarod time-to-fall scores over trials can exhibit significant variability, requiring multiple trials by multiple mice to discern relatively subtle differences in motor learning. Having more robust measures of motor performance during rotarod trials could help discern motor behaviors better, potentially reducing the number of trials and mice used.

In this context, in order to improve upon these motor performance assessments, we drew upon two simple principles: a) motor activity is as detailed or information-rich as the resolution power of the means employed for its observation, and b) in normal conditions, movement, including its fine structure (or sub-structure), is a reproducible activity subject to practice and consequent learning and these aspects are amenable to quantification. Therefore, we set out to extract and characterize further motor features from *within* a mouse’s first rotarod session and from subsequent trials in order to quantitatively evaluate motor behavior in depth.

Intra-session assessment of motor function during a rotarod test is feasible via the video recording of mice followed by scoring via direct observation of mouse limb movements^8^. More precise assessment of limb movements can be achieved by tracking the vertical paw position of the mouse^9^, whereby the average paw position height increases with rotarod practice. Capitalizing upon these possibilities, we have developed a method that utilizes video-tracking of the right hind paw of the mouse to define and evaluate multiple unique features that characterize motor performance in greater detail. Our results show that vertical and horizontal smoothness of paw speed as measured by spectral arc length is inversely correlated with rotarod motor performance scores for any one of 3 trials. Vertical paw position irregularity over time (measured as approximate entropy) was positively correlated with rotarod scores, but this occurred only in the first trial. In contrast, horizontal paw approximate entropy exhibited an opposite correlation. Smoothness and approximate entropy indices exhibited significant correlations with rotarod scores in a subset containing as few as 9 mice and these correlations were strengthened by increasing mouse numbers. Horizontal approximate entropy in the initial seconds of the first trial predicted subsequent rotarod scores at the completion of the trial, suggesting that this parameter is sensitive to innate or untrained mouse paw movement capacities that determine motor performance. Analogously to rotarod scores, intra-session measures robustly reflected group differences. Unlike the observations made for rotarod scores, the intra-session measure of paw vertical movement smoothness, vertical speed in the first 16 s and horizontal median absolute deviation increased in the second trial, suggesting that these measures are sensitive to single-trial motor learning. Using a support vector machine classifier, it was corroborated that numeric feature values at the end of the first trial are distinct from those at the beginning, indicating that these features are responsive to mouse motor behavior, as it changes over the time course of an accelerating rotating rod. Analysis of mice injected with one dose of baclofen insufficient to impair rotarod performance, revealed that the method allows for the characterization of minimally impaired movement.

## Materials and methods

### Rotarod performance test

All assays were conducted blindly to mouse weight or baclofen treatment. A standard accelerating rotarod apparatus (Harvard Apparatus) with capacity for simultaneously testing up to 5 mice was employed. 32 wild-type mice (totaling 21 male and 11 female littermates for all experiments) were used after being labeled with metal ear tags, allowing for individual identification. These mice were divided as follows: a) 22 mice (11 male and 11 female) were used for first trial rotarod analysis. From these mice, a first group containing 9 mice (4 male and 5 females) also underwent a second and third rotarod trial with video analysis of paw position; b) the remainder 10 mice were injected with baclofen and compared to weight-matched uninjected controls. Because mouse weight can impact rotarod performance^10,11^, a subset of the 22 mice cited above with the most divergent weights (the 6 highest and 6 lowest-weight of the 22) were divided into two groups for additional comparison: a) larger-weight (more than 25 g; 30 ± 1.3 g, n = 6) and lower-weight (20 g or less; 16.6 ± 1.1 g, n = 6); *p* < 0.0001 for the difference in weights, two-tailed unpaired student’s *t*-test.

Rotarod initial rotation velocity was 4 RPM and accelerated by one RPM every 8 s, to a maximum of 40 RPM. As is standard in a rotarod apparatus, a falling mouse landed on a platform that was connected to a timer, which reported the time spent on the rotarod (time-to-fall) or raw rotarod score. A second and third trial were conducted 3 to 4 h and 24 h after the first trial. A video camera placed in the same horizontal plane as the rod recorded the rotarod session from the rear of the mice for subsequent paw tracking analysis.

### Video mouse paw tracking

**Figure 1:**
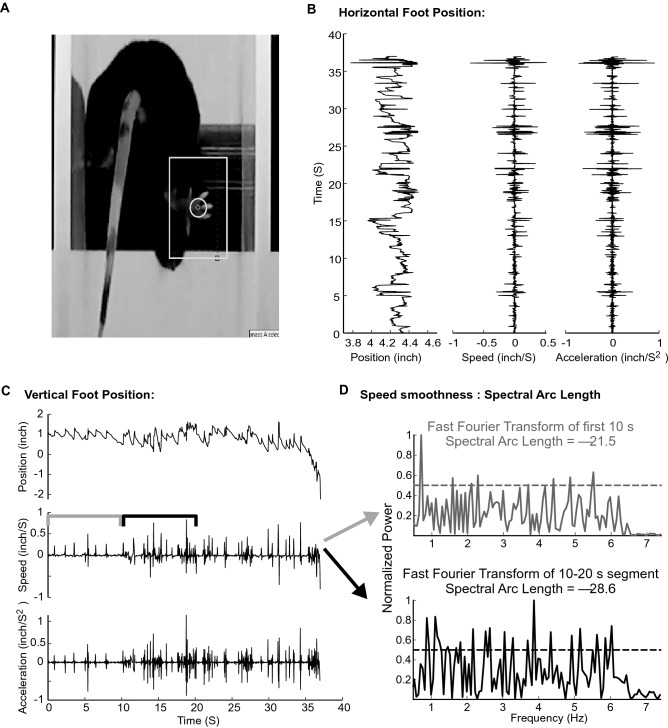
Mouse undergoing a video-rotarod trial. (**A**) Mouse complying with the movement of a rotating rod. A gray colored rectangle outlines the region of interest around the paw (or foot) that the program utilizes to locate serial paw positions. (**B**) Horizontal paw position, speed and acceleration of the right hind limb paw of a mouse over the course of its first rotarod trial. (**C**) Vertical paw position, speed and acceleration of the right hind limb paw of a mouse over the course of its first rotarod trial. Grey and black lines bracket the data analyzed in similarly colored graphs in (**D**). (**D**) Example normalized spectrograms illustrating the computation of spectral arc length to measure movement smoothness. Paw speed earlier in the trial (grey), which undergoes more regular speed deflections, is associated with a normalized spectrogram (grey) with less prominent peaks and thus with a smaller absolute spectral arc length. A less negative, closer to zero, magnitude of this value indicates greater smoothness.

The softwares “Tracker: Video Analysis and Modeling Tool” (https://physlets.org/tracker/) and Kinovea (https://www.kinovea.org/) were used to track the right hind limb paw of a mouse on the rotarod apparatus (Fig. 1A) as previously reported^9^. There was no appreciable difference in the results obtained with either program. In brief, a rectangular region of interest including the target object (in this case, the relatively pale paw of the mouse visible against the darker background of mouse body and rod) was used by the program to track the paw in continuous video frames and chart its course over two-dimensional space and time. All tracking was supervised offline to provide human confirmation that the program faithfully followed the right hind limb paw. Each tracking analysis was referred to a pre-trial distance calibration (in inches). The junction of the lower border of the rotating rod and the left partition was assigned coordinate [0,0] and the tracked paw position was assigned coordinates relative to this reference point. Values corresponding to the x- (horizontal) axis coordinates (Fig. 1B) and y-axis coordinates (Fig. 1C) of the paw were exported to an Excel spreadsheet for further analysis. Videos were recorded at either 30 Hz or 15 Hz prior to analysis, with no appreciable difference detected between the two recording rates.

### Video-rotarod intra-session features derived from mouse paw coordinates

Paw position data in Excel spreadsheets were imported into MATLAB (The MathWorks, Inc.) for analysis with previously available MATLAB functions. Data was resampled at 15 Hz (using MATLAB function resample) prior to analysis to ensure a common sampling rate. Putative motor-performance-relevant features were generated based on the following rationale : *a) The mean vertical position* (y-axis coordinate) of a paw may reflect an attempt to stay on the rotating rod and should be lower if the paw slips or the mouse is otherwise unable to maintain the pace of the rotation*. b) The mean horizontal position* (x-axis coordinate) of a paw may be responsive to a mouse attempt to balance itself on a rotating surface by widening its stance or gait. *c) The mean vertical speed and acceleration* of paw movement may be negative if there are more rapid downward paw slips as compared to the even upward rotation of the rod and the balanced paw moving with it or reaching for it. The absolute speed of both upward and downward motions was considered unless otherwise noted. *d) The vertical variance* of the paw position may reflect that a mouse either made a variety of paw positioning attempts both of small and large amplitude or that approximately the same uniform paw reach amplitude was observed. *e) The vertical approximate entropy* measured the regularity of paw movement over time. Approximate entropy was used to evaluate the likelihood, in a time-series, that similar patterns repeat across time. In this analysis, a sine wave time-series would display low approximate entropy whereas an irregular trace would exhibit higher approximate entropy. For example, a metronome-like forward movement of the paw every second resulting in a sinusoidal time-series trace would exhibit low entropy, whereas a forward movement at varying time intervals would yield high approximate entropy. This measure has been previously used to measure the regularity of rodent gait patterns on a flat moving surface in a treadmill task^12^. *f) Spectral arc length* measured the smoothness of paw speed as it adapted to the accelerating rod. Spectral arc length has been used previously in human motor studies to characterize smoothness of movement^13–15^. In brief, this measure utilizes a fast Fourier transform of the speed and a normalized speed spectrogram (Fig. 1D). In this analysis, less smooth speeds are characterized by the appearance of multiple prominent speed frequencies in the spectrogram. The arc length is the length of the spectral trace, and its numeric value is calculated by a sum of the integral at each point. Since greater spectral prominence and a resultant longer spectral trace reflects less smooth movement, its value is conventionally multiplied by − 1 to represent smoothness. This sum thus represents the smoothness of the corresponding speed, where values closer to zero indicate smoother velocities over time. A similar spectral arc length can be calculated for acceleration. g, h, i) The horizontal mean speed, variance, approximate entropy and spectral arc length for speed and acceleration were selected with the same rationale as the corresponding vertical coordinate parameters. The overall rationale for the horizontal features was that it may measure the mouse attempts to balance itself by adjusting the width of its gait, whereas the rationale for the vertical features was that it may measure the mouse attempts to stay on the accelerating rotation, with vertical paw slips reflecting when it is failing to remain on the rod. The value of the following features was also assessed: a*) Maximum vertical position* exhibited a maximum for values at the rotating rod’s upper border and was considered non informative. *b) Maximum horizontal position* displayed a maximum for values at the right sided partition and was not deemed informative. *d) Minimum vertical position* was not included as a measure of global rotarod performance as it would merely report the position of a mouse after falling. However, minimum *vertical position for each one-second time interval (or bin)* was utilized for assays involving the support vector machine classifier (SVM). *e) Downward paw speed* was used for assays involving the support vector machine classifier.

### Baclofen administration

Baclofen is a widely used drug with several effects on the motor system that impact performance and can exert sedation at elevated doses^16^. We used it at low-dose to test whether a small degree of change in motor performance (i.e., not associated with rotarod-detectable motor impairment) could be characterized by the method. Littermate mice were separated from the same group that we studied and injected intraperitoneally with 5 mg/kg. Duration from injection to testing was 30 min.

### Support vector machine (SVM) based behavior classifier

We examined the change in the magnitude of all the above features between the first and the last twenty seconds of the rotarod task with the expectation of detecting acceleration-dependent parameters. Since the rod accelerates soon (8 s) after starting the task, the responsiveness and evolution of the first trial intra-session features were evaluated over the course of the trial. The one-second time intervals selected for the measurement of intra-session parameters could introduce extreme outlier values as a consequence of their brief duration since the mouse is incessantly moving in a priori-unpredictable directions to comply to the task. Therefore, the temporal evolution of calculated one-second intra-session features was analyzed using a modification of the MATLAB “*smooth*” function to reduce the influence of extreme values. This function applied local regression using weighted linear least squares, assigning zero to any data that lie outside six mean absolute deviations and allowed for the generation of temporal trajectories less influenced by such values. A best-fit trend line (a 3rd degree polynomial) was also used to independently visually evaluate the fidelity of this procedure. For every second on the rotarod, and for both vertical and horizontal positions, we computed the features and determined their median value for that second if there was more than one derived value (see Methods 2.3). The number of features were limited to a maximum of 6 (3 obtained from the y-axis, with another simultaneously measured 3 from the x-axis) to avoid overfitting the data. This yielded two classes of values for each feature selected, corresponding to early rotarod and late rotarod performance, with each class of values containing 20 observations corresponding to every second in a 20 s task interval. We utilized SVM classifier functions in MATLAB as described^17^. In brief, the SVM classifier calculates a plane or decision boundary that optimizes a separation between classes across several computational cycles. The decision boundary shapes tested included linear, Gaussian or polynomial (2nd, 3rd or 4th order) kernels. In order to establish the optimum kernel, a tenfold cross-validation for each kernel determined the misclassification rate. The kernel with the lowest misclassification rate was selected. Additionally, other optimized hyperparameters included kernel scale (whereby a higher scale examined a narrow region of points near the decision boundary resulting in a less sharply defined boundary), box constraint (which was susceptible to decision boundary-outlying values and thus minimized overfitting) and standardization (as all feature values were normalized by subtraction from their mean and division by their standard deviation). For each subsequent cycle of the SVM classifier and cross-validation, hyperparameters were successively adjusted to achieve optimal separation between the two classes. The classifier yielded Receiver Operating Characteristic (ROC) curves as the Area Under Curve (AUC) and average misclassification rate. An AUC of 0.5 indicated no difference between the two classes while an AUC of 1 indicated that the two testing groups separated into two distinct clusters. A misclassification rate of 0.5 is associated with equal chance of any data point belonging to either class. A misclassification rate less than 0.05 indicates a less than 5% probability that a randomly selected data point is assigned to the wrong group. In other words, there is minimal overlap of the two testing groups.

For multidimensional data (including each dimension of a measured mouse paw parameter) in baclofen-treated mice, principal component analysis (PCA) was employed to reduce the number of dimensions and allow visualization of differences between baclofen-treated and control mice. In brief, PCA was used to geometrically project data onto dimensions called principal components, aiming to find the best description of any clustering in the data. For example, a two-dimensional ellipsoid cluster of points with a positive slope of 1 for its best-fit line can be reduced with least loss of information to a single dimension by determining its orthogonal projections on the best-fit line. In other words, the diagonal best-fit line forms a new axis (Principal Component 1), and the distribution of values on it best reflect the spread of two-dimensional data in one dimension with the least loss of information.

### Statistics

Statistical and other comparisons employed: a) Correlation analyses for two variables: *p*-values were obtained as an output of the ‘*corr*’ MATLAB function (for linear or rank correlation). A *p*-value < 0.05 indicates rejection of the null hypothesis that no correlation (where slope = 0) exists. Spearman’s correlation coefficient and the associated *p* value assessed the association between the rotarod time-to-fall and various parameters for the first, second and third trial; b) Two-tailed unpaired Student’s *t*-tests were applied to one dimensional comparisons between high-weight and low-weight groups, and control and baclofen treated mice for their first rotarod trial features; c) Paired Student’s *t*-tests were utilized to examine changes between the first and second trials; d) The MANOVA-Wilks test was employed to examine differences between control and baclofen injected mice for the three-dimensional data derived from the first three principal component analyses. Except where indicated, statistical results are expressed as mean ± standard error of the mean (S.E.M.). All of the comparisons described in the text in narrative form were statistically significant (*p* < 0.05) unless otherwise stated.

### Animal use approval

Institutional Animal Care and Use Committee of UT Southwestern Medical Center.

## Results

### Vertical paw position features in the first video-rotarod trial

**Figure 2:**
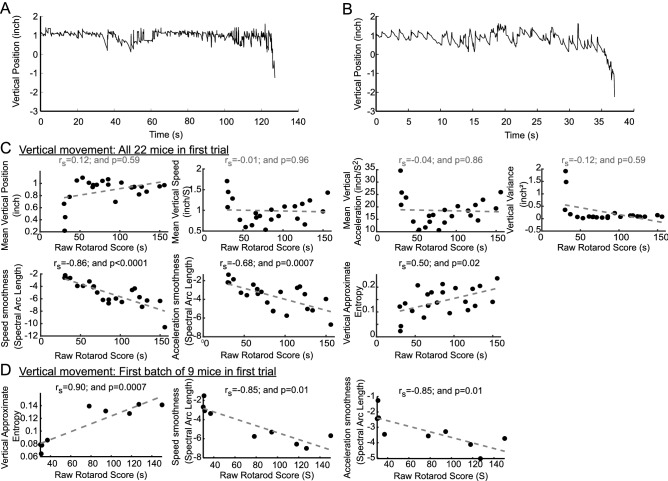
Relationship of first trial vertical paw position intra-session features with rotarod scores across mice. (**A**,**B**) Representative vertical paw position changes over time for mice with greater (**A**) and smaller (**B**) rotarod scores. A regular “saw-tooth” pattern (indicating a lower approximate entropy) characterizes mice with lower rotarod scores in B compared to mice in A. (**C**,**D**) Scatter plots for intra-session features with their best fit line. Spearman correlation coefficient rho (r_s_) and a measure of statistical significance (“p”) are provided above each plot.

The temporal evolution of the vertical position of the right hind limb paw reflected the expected fact that some mice performed better (with ‘better’ defined, as in common usage, to indicate staying on the rotating rod longer) than others (Fig. 2A,B, respectively), as normally seen in motor tasks performed by genetically identical or similar individuals. When the raw rotarod score for each mouse (or, as above, the total duration on the rotarod) was compared with each set of intra-session features (see Methods 2.3).

Approximate entropy was correlated with rotarod scores (Fig. 2C), reflecting that mice that exhibited less regular paw movements over time in the vertical axis stayed on longer on the rotating rod.

The smoothness of paw speed and acceleration were measured using spectral arc length. Paw smoothness for vertical movements was inversely correlated with raw rotarod scores. The mean height of the paw, vertical speed, acceleration and variance of vertical movements were not correlated with total time on the rotarod.

In order to characterize parameters robust enough for capturing motor behavior from smaller mouse groups, we asked if features deemed significant in the analysis above would also be significant for a subset of only 9 mice. We found that vertical paw smoothness of speed and acceleration and approximate entropy were also significantly correlated with time-to-fall for these 9 mice (Fig. 2D).

### Horizontal paw position features in the first video-rotarod trial

**Figure 3:**
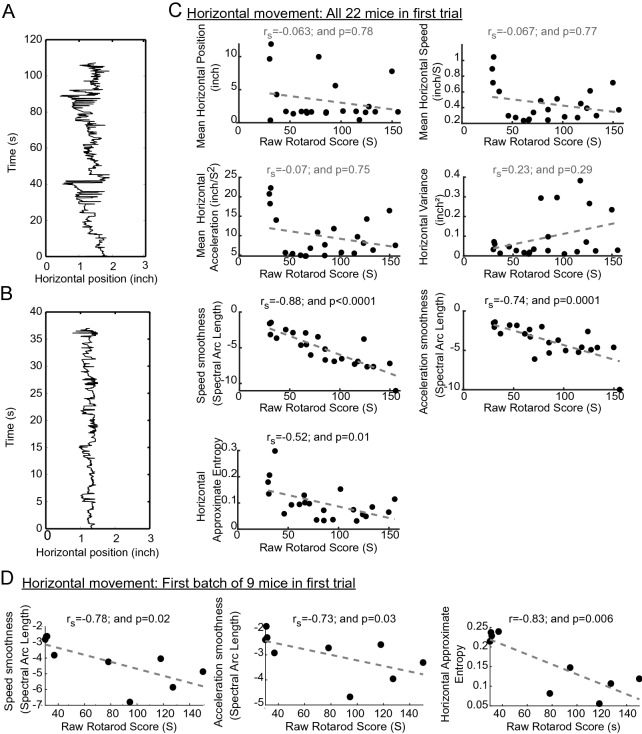
Relationship between first trial horizontal paw position intra-session features and rotarod scores across mice. (**A**,**B**) Representative horizontal paw position changes over time for mice with greater (**A**) and smaller (**B**) rotarod scores. (**C**,**D**) Scatter plots for intra-session features with their best fit line. Spearman correlation coefficient rho (r_s_) and a measure of statistical significance (“p”) is provided above each graph.

We examined horizontal paw position for mice with different rotarod scores (Fig. 3A,B) in relation to intra-session features. In contrast with paw height, which showed no correlation with rotarod scores, approximate entropy was *negatively* correlated with these scores (Fig. 3C). This reflected the fact that mice exhibiting more regular horizontal movements over time achieved greater rotarod scores. Similarly to vertical paw movements, less smooth horizontal paw speed and acceleration were associated with greater rotarod scores and these parameters were also correlated for a the smaller subset of mice (Fig. 3D).

### First trial early predictors and task difficulty-dependent features

**Figure 4:**
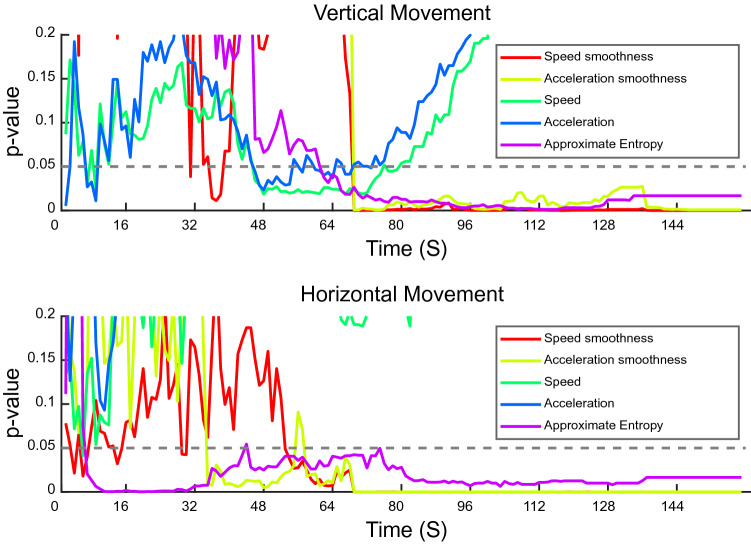
First trial early predictors and task difficulty dependent features. Vertical and horizontal movement features examined over time of rotarod performance. Each parameter was examined for correlation with final rotarod scores for each increasing time segment from the start of the trial. The *p* value for this correlation analysis for each time segment is depicted on the y-axis.

We hypothesized that one or more quantifiable features observed early in the first trial, while the mouse was displaying its initial interactions with the motor task and the motor demand was thus least, may predict rotarod performance. In contrast, any feature(s) that may require quantification over a longer time to achieve correlation with rotarod scores would depend upon a mouse increasing its effort as the motor task difficulty increased. Because the rotarod accelerated every 8 s, with mouse fatigue likely increasing every second, feature values were computed for every cumulative 1 s time segment (i.e., for the first 8, 9, 10, 11, 12, etc. s) and a *p* value was calculated by examining the correlation of the averaged feature value for that initial time segment with the rotarod score Two categories of predictors were observed based on these correlations: (i) Early predictors: Horizontal approximate entropy calculated in the first 16 s on the rotarod was sufficient to demonstrate an inverse correlation with rotarod scores (Fig. 4). Very early vertical speed and acceleration measures indicated a correlation with rotarod scores. (ii) Late predictors: The spectral arc length for both vertical and horizontal speed and acceleration for the first ~ 75 s also sufficed to predict rotarod scores (Fig. 4). Of note, all mice tested spent at least 30 s on the rotarod.

### First trial features compared between mice groups and with second trial

**Figure 5:**
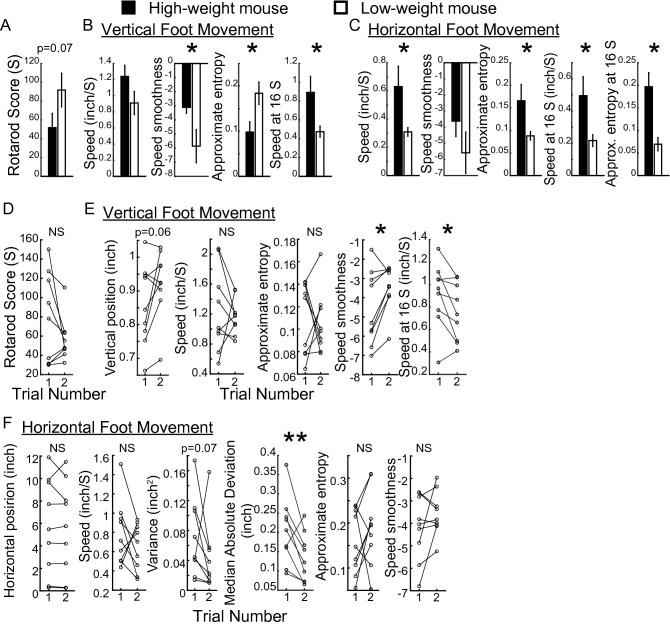
Comparison of first trial features between mice groups and with second trial. (**A**–**C**) Rotarod score (**A**) and vertical height and horizontal (**B**,**C**) intra-session features in smaller-weight (n = 6) and greater-weight (n = 6) mice. Bar charts illustrate means and error bars represent standard error of mean for rotarod score and intra-session features for vertical paw position. * = *p* < 0.05, ** = *p* < 0.01, (unpaired Student’s *t*-test). (**D**,**E**) Change between the first and second trial in rotarod scores (**D**) and vertical (**E**) and horizontal (**F**) intra-session features in all mice tested (n = 9). * = *p* < 0.05, ** = *p* < 0.01 (paired Student’s *t*-test).

Lower-weight mice performed on average better than heavier mice on the rotarod task but this was not significantly different for the mice tested (n = 6 in both groups; Fig. 5A). As hypothesized above, multiple features including approximate entropy and smoothness measures differentiated lower-weight from heavier-weight mice (Fig. 5B,C). Mean speed for the first 16 s of a trial was significantly lower for both axes for the lower-weight mice. This indicates that these features are relevant in discerning whether groups of mice differ in motor ability.

Following the first trial, the rotarod test score did not demonstrate a consistent increase for all tested mice (Fig. 5D). However, intra-session features in the second trial such as smoothness of vertical speed and vertical speed in the first 16 s and displayed significant changes for all mice (Fig. 5E). Horizontal foot variance did not exhibit any significant changes, but horizontal median absolute deviation (a measure that is less influenced by time-series outliers compared to variance) significantly decreased in the second trial (Fig. 5F).

### Paw position features in second and third video-rotarod trials

**Figure 6:**
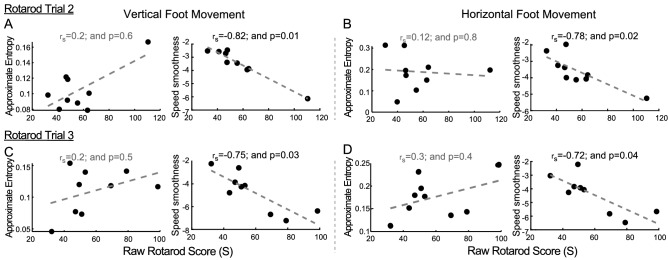
Relationship between second and third trial vertical and horizontal paw position intra-session features and rotarod scores. (**A**,**B**) Relationship of vertical and horizontal paw position approximate entropy and speed smoothness with rotarod scores across all mice during their second trial. (**C**,**D**) Relationship of vertical and horizontal paw position approximate entropy and speed smoothness with rotarod scores for the mice during their third trial. Graphs are scatter plots for intra-session features with their best fit line. Spearman correlation coefficient rho (r_s_) and a measure of statistical significance (“p”) is provided for each graph.

The correlation between intra-session features and rotarod performance in a subsequent rotarod trial 4 h later (second trial) was also examined (Fig. 6A). Because sleep influences motor learning or plasticity^18^, we also analyzed whether the features under examination correlated with rotarod performance 24 h later, following sleep (third trial) (Fig. 6B). This yielded no correlation when comparing rotarod score with approximate entropy. In contrast, smoothness indices for vertical and horizontal speed were correlated with rotarod score for the second and third trials.

### Support vector machine analysis of motor features at start and end of first rotarod trial

**Figure 7:**
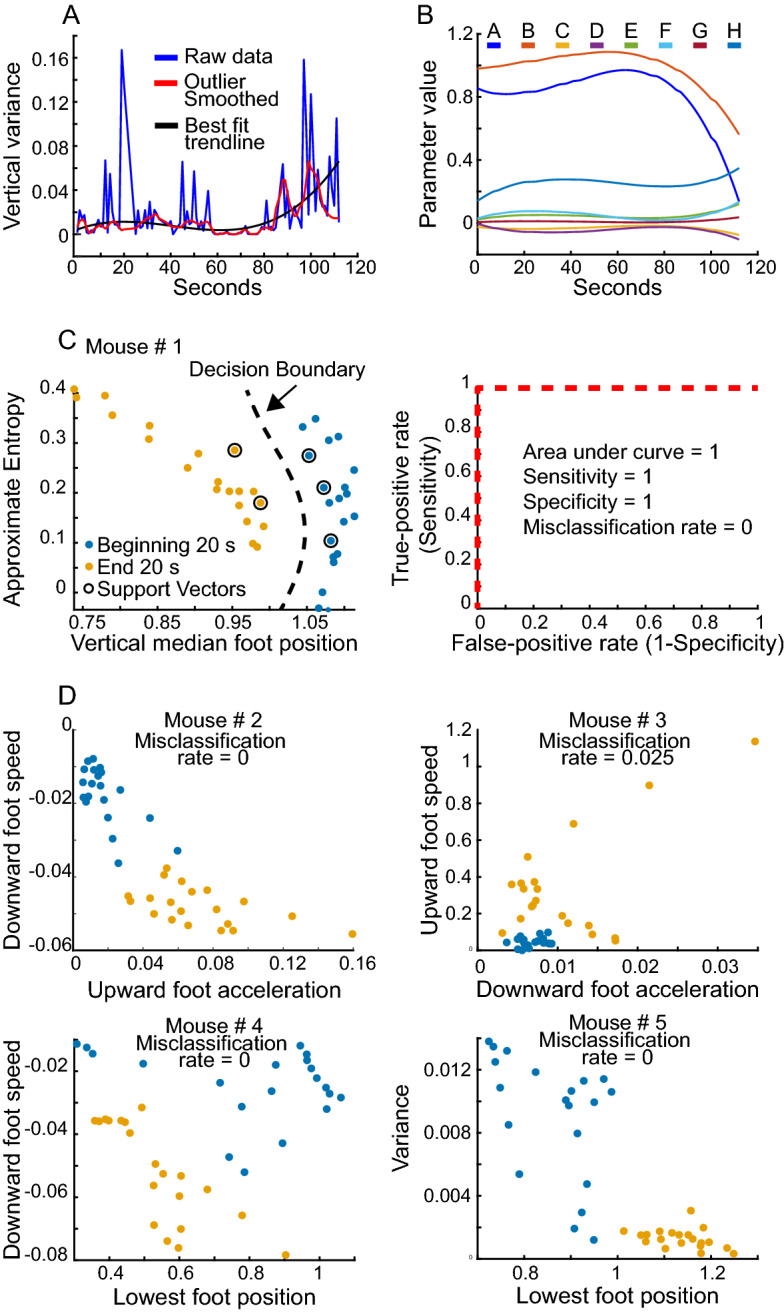
Temporal profile and support vector machine analysis of intra-session features. (**A**) Temporal profile of variance of vertical position of paw calculated for 1 s time intervals (blue line). The red line arises from the treatment of the unmodified data with a function that assigns a value of zero to values that deviate 6 or more standard deviations from the mean. A 3rd degree polynomial least-squares best-fit line (red) illustrates trends across seconds. The last 5 s have been omitted to avoid including values corresponding to a falling mouse. (**B**) Best fit trend lines for several parameters illustrate a change in the last 20 s before the mouse falls off the rotarod. The last 5 s have been omitted to avoid including mouse falls. A = Lowermost paw position, B = median paw position, C = Downward paw speed (negative values indicate downward movement), D = Downward paw acceleration (negative values indicate downward movement), E = Upward paw speed (negative values indicate downward movement), F = Upward paw acceleration (negative values indicate downward movement), G = Variance of the vertical position of the paw, H = Approximate entropy of the vertical position of the paw. (**C**) Support Vector Machine (SVM) classifier. *Left panel*: Two-dimensional (2D) example scatter plot of vertical paw position and approximate entropy calculated for the first 20 one-second time intervals contrasted with 20 one-second time intervals at the end of the rotarod session. Each dot corresponds a 1-s time interval or bin. The last 5 s have been omitted as above. The data-driven decision boundary distinctly separates the two classes of mice. *Right panel*: Receiver operating characteristic (ROC) curve for classifying the first and last 20-s time-bin data in separate clusters for the mouse depicted in the left panel. The classifier analysis was conducted in 6-dimensional space on vertical and horizontal paw speed, acceleration, and variance of paw position calculated for 21-s time bins. (D) Scatter plots for different mice illustrating separation of features corresponding to the first and last 20 s of a rotarod session. The misclassification rate (MR) calculated from an SVM classifier is indicated for each graph.

As described in Methods, the mouse data were pre-processed to limit the impact of extreme outliers (Fig. 7A) and best-fit trend lines were examined visually to confirm trends over time (Fig. 7B). In the 5 mice that exhibited greater rotarod scores, we compared the magnitude of principal-component-derived features in the first 20 s with the last 20 s of the mouse on the rotarod using a support vector machine-based behavior classifier. The classifier addressed whether the two classes (i.e., feature data from first 20 s and from the last 20 s) belonged to the same or different data clusters. For these 5 mice (Fig. 7C,D), AUC scores were 1, with misclassification rates less than 0.05. The parameters that best separated the two classes varied across mice, suggesting that each mouse may slightly vary in its adaptation to the accelerating rod. These results indicate that the first trial intra-session features change over the course of the rotarod trial and reflect the necessary change in mouse motor behavior during the task.

### Characterization of baclofen-induced motor changes

**Figure 8:**
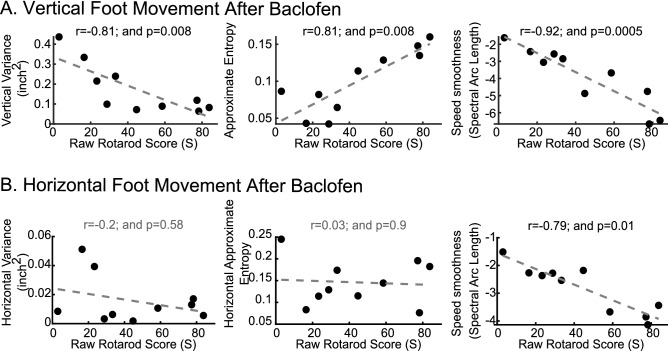
Relationship of paw features with first trial rotarod scores following baclofen administration. Scatter plots for y-axis (**A**) and x-axis (**B**) intra-session features with their best fit line. Spearman correlation coefficient rho (r_s_) and a measure of statistical significance (“p”) are provided above each plot.

**Figure 9:**
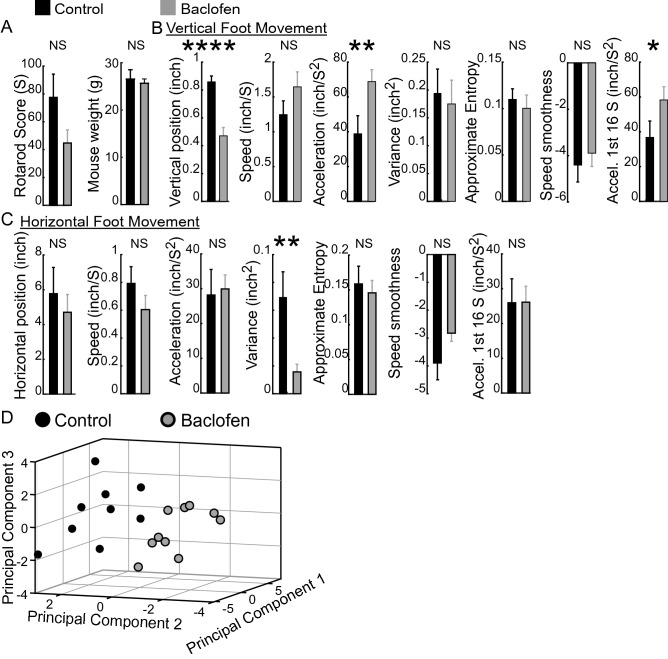
Impact of baclofen on first trial features. (**A**–**C**) Rotarod score and mouse weight (**A**) and vertical height and horizontal (**B**,**C**) intra-session first trial features in control (n = 9) and baclofen-injected mice (n = 10). Bar charts illustrate means; error bars represent S.E.M for rotarod score and intra-session features for right hind-limb paw position. Significant changes in some intra-session features are notable despite lack of change in rotarod scores. (**D**) Scores derived from principal component analysis (PCA) reveals distinct distributions (*p* < 0.0001, MANOVA-Wilks test) for baclofen-injected (n = 10) and control mice (n = 9). Each dot represents one mouse. Values along an axis represent PCA scores for that principal component. A mouse can be represented in multi-dimensional space with each intra-session feature comprising one dimension. Dimensionality reduction with PCA reveals separate groups. * = *p* < 0.05; ** = *p* < 0.01; **** = *p* < 0.0001, (unpaired Student’s *t*-test).

We also asked if our method could detect minimal (i.e., not apparently visible) motor deficits in mice undifferentiated by rotarod time-to-fall assay. We thus examined mice injected with 5 mg/kg baclofen, a dose below that shown to cause deficits in rotarod scores^16^. We first examined if baclofen-injected mice exhibited intra-session features that correlated with rotarod scores as described above for control mice above. Figure 8 illustrates the relationship of paw features with first trial rotarod scores following baclofen administration. We found that paw speed smoothness in both axes and vertical variance and approximate entropy correlated with rotarod scores. This result is the more relevant because baclofen-injected mice had weights similar to control mice (Fig. 9A) and did not exhibit any significant difference in rotarod scores relative to control (control: 77.5 ± 16.8 s, n = 9; baclofen-treated: 44.8 ± 9.5 s, n = 10. *p* = 0.08). In contrast, the intra session features of vertical position and acceleration and horizontal foot variance were significantly impacted in the baclofen-injected mice (Fig. 9B,C). Strikingly, vertical acceleration measured in just the first 16 s was sufficient to differentiate the two groups. Principal component analysis incorporating a combination of all the features revealed different principal component score distributions (Fig. 9D, p < 0.0001, MANOVA-Wilks test), illustrating the sensitivity of the measures in making group motor differences manifest after a single rotarod trial.

## Discussion and conclusions

Our study is, to the best of our knowledge, the first application of the spectral arc length measure to study animal movement and the first use of several paw movement measures to discern motor behavior relevant parameters in initial motor trials.

### Significance of paw speed uniformity or smoothness

Our findings show that the average smoothness of paw speed in vertical and horizontal axes inversely correlates with rotarod performance (scores) for all 3 initial trials. Thus, speed smoothness can be utilized as an intra-session parameter that reports paw activity relevant to the rotarod scores that will be ultimately achieved. There are two possible and opposite interpretations of this inverse relationship between average paw speed smoothness and rotarod scores, which attribute the relationship to either: (1) *Mouse motor ability*: a constantly changing and less smooth paw speed may stem from the ability of a mouse to keep adapting to a rotating surface and comply with the task, whereas a fixed or invariant paw speed may result in a greater probability of losing balance on the accelerating rod. Or (2) *Longer duration on the rotarod*: A mouse that stayed longer on the rotarod experienced a more difficult task due to the faster rotarod rotation, which may result in increasingly disorganized or chaotic paw steps at the end, thus drawing average paw smoothness towards lower values. In favor of the second interpretation, for an identical task difficulty in the first trial (i.e., the first 30 s with all mice staying on the rod), Fig. 4C illustrates that paw speed smoothness did not correlate with rotarod scores. This suggests that the reduced speed smoothness is the result—rather than the cause—of longer mouse duration on the rotarod. Therefore, speed smoothness may be used as a surrogate or predictor of rotarod scores in initial trials. However, for each mouse, vertical speed smoothness increased from the first to the second trial (Fig. 5E) and this included mice with lower rotarod scores. This indicates the potential of this measure to anticipate motor changes in one-trial learning. This increase in smoothness or uniformity took place despite a lack of regularity of changes in rotarod scores from the first to the second trial (Fig. 5D). Therefore, paw speed smoothness is influenced by at least two factors: a) Within the same mouse, it may reflect a modulable process susceptible to one-trial learning. And (b) across mice (Figs. 2C and 3C), inter-mouse variability in paw speed smoothness may simply stem from the amount of time that each mouse spends on the rotarod, with proportionate demands on paw movement as that time varies. This across-mice relationship between speed smoothness and rotarod scores (Figs. 2C and 3C) is preserved following baclofen administration, suggesting that this is a robust measure capable of identifying motor differences within groups.

### First trial predictors of motor performance

In contrast with paw speed smoothness approximate entropy was numerically related to rotarod scores only for the first trial. This may be due to inherent paw placement variability upon first exposure to a motor task. For example, mice that tend to vary their stance across time more (i.e., those which can display greater horizontal paw regularity of placement over time) could balance better on the rotarod and thus achieve greater rotarod scores. Learning from the first trial could effect change by expanding paw placement possibilities such that approximate entropy ceased to be as relevant in subsequent trials. In support of this, practice of a motor task decreases the variability of relevant movements^19^.

Remarkably, horizontal approximate entropy during the first few seconds and very early paw vertical speed and acceleration were sufficient to predict first trial rotarod performance (Fig. 4). This may be accounted for by two phenomena: a) A slower speed may reflect a more deliberate paw placement and regularity over time, resulting in greater rotarod scores. Or (b) faster speeds or less regular placement could stem from paw slippage, resulting in reduced rotarod scores. In either case, measuring these parameters very early in the first trial sufficed to anticipate motor performance in that trial. This suggests that these features may be susceptible to innate or untrained paw movement ability or propensity prior to motor learning. Baclofen administration, used here to model a subtle motor deficit, was associated with reduced horizontal variance and increased vertical acceleration, including those measured in the first 16 s, thus suggesting the possibility of the detection of group motor differences after or even within the first rotarod trial.

### Inverse relationship of vertical and horizontal approximate entropy

Approximate entropy for both x-axis and y-axis coordinates were correlated with motor performance, but in opposite sense for the x-axis relative to the y-axis paw positions. This may indicate that paw movements or excursions in one axis occur at the expense of the other. For example, a mouse attempting to balance itself by broadening its gait would move its paw horizontally more than vertically, whereas a mouse aiming to move ahead would move its limb upwards on the rod rather than horizontally. If a mouse attempts to maintain balance by serially removing and placing its paw back in the same x-axis location (i.e., following a more regular, metronome-like horizontal movement as assayed by approximate entropy), the regularity of paw vertical positioning may decrease, resulting in greater approximate entropy for the vertical paw position. A derivative question (outside the scope of this study) is whether rodents maintain balance on the rotarod via horizontal gait-adjusting movements instead of the way they would comply with an inclined treadmill, which would require forward and upward movement of the paw.

### Limitations and conclusions

The tracking of markers placed on a mouse can interfere with natural movement^20,21^. Our study attempts to circumvent this via the markerless tracking of mouse movement. This study was limited in that it tracked a single paw position. Methodologies based on deep learning have the capacity to track multiple body regions and their relation to each other without markers, thus allowing the characterization of the pose of the mouse^22^. Markerless tracking offers a better approach to evaluate theories of natural movement such as those that rely on the motor learning of a sequence of movements^23^, with participation from diverse central and peripheral nervous system regions, some of which may enable aspects of motor learning.

Another aim of this study was the identification of robust intrasession parameters to characterize motor differences using as fewer mice and fewer trials as possible. We utilized 22 mice to examine the relationships between rotarod time-to-fall in the first trial and intra-session parameters. We then asked if these relationships were robust enough to allow for the using of fewer mice and found significant differences in comparing motor behavior groups. However, the use of fewer mice may likely result in the loss of less robust but nevertheless meaningful differences between groups. Thus, having a relatively lower number of mice for analysis is a likely limitation of this study. Similarly, whereas all mice could spend at least 30 s on the rotarod, there were fewer mice that spent over 2 min, likely rendering early predictors of final performance (Fig. 4) more reliable.

A striking feature in our assay was the robustness of spectral arc length in capturing both intra-session motor performance and motor learning across sessions. Spectral arc length has been used in man to characterize movement^13^. It thus possible that, upon motor learning, increased coordination of agonist and antagonist muscles leads to smoother movement reflected in spectral arc length.

Our findings reveal multiple motor performance-relevant intra-session features obtained from video-rotarod recordings by the tracking of the cartesian coordinates of the mouse paw. These features are those which: (i) Report inherent paw movement predilections in the first few s (*horizontal approximate entropy*, *vertical speed and acceleration*) and during (*horizontal approximate entropy and paw movement smoothness*) the first exposure to a motor task; (ii) can reflect one-trial learning (*vertical speed smoothness and early vertical speed and horizontal median absolute deviation*); and iii) correlate with rotarod scores across the initial trials (*vertical and horizontal speed and acceleration smoothness)*. We expect that, by expanding the methods for the analysis of initial rotarod trials as described, variations in early motor learning and the motor performance of mutant or pharmacologically treated mice will be more amenable to robust quantitative assessment. Further, if the expected learning can be estimated from features observed during—or extracted from—the early performance of a repetitive task, interventions that enhance or interfere with such learning may be administered before learning completion.
