# Author Correction: Quantification of early learning and movement sub-structure predictive of motor performance

**DOI:** 10.1038/s41598-021-99461-z

**Published:** 2021-09-29

**Authors:** Vikram Jakkamsetti, William Scudder, Gauri Kathote, Qian Ma, Gustavo Angulo, Aksharkumar Dobariya, Roger N. Rosenberg, Bruce Beutler, Juan M. Pascual

**Affiliations:** 1 Rare Brain Disorders Program, Department of Neurology, The University of Texas Southwestern Medical Center, 5323 Harry Hines Blvd., Mail Code 8813, Dallas, TX 75390-8813 USA; 2 Center for the Genetics of Host Defense, The University of Texas Southwestern Medical Center, Dallas, TX USA; 3 Department of Physiology, The University of Texas Southwestern Medical Center, Dallas, TX USA; 4 Department of Pediatrics, The University of Texas Southwestern Medical Center, Dallas, TX USA; 5 Eugene McDermott Center for Human Growth and Development/Center for Human Genetics, The University of Texas Southwestern Medical Center, Dallas, TX USA

Correction to: *Scientific Reports* 10.1038/s41598-021-93944-9, published online 13 July 2021

The original version of this Article contained errors.

In the legend of Figure 2,

“A larger distribution amplitude (i.e., an increased variance) in a regular “saw-tooth” pattern (indicating a lower approximate entropy) characterizes mice with lower rotarod scores in B compared to mice in A. (**C**) Scatter plots for intra-session features with their best fit line.”

now reads:

“A regular “saw-tooth” pattern (indicating a lower approximate entropy) characterizes mice with lower rotarod scores in B compared to mice in A. (**C**,**D**) Scatter plots for intra-session features with their best fit line.”

In the legend of Figure 3,

“(**A**,**B**) Representative horizontal paw position changes over time for mice with greater (**A**) and smaller (**B**) rotarod scores. A broader distribution of amplitudes (indicating greater variance) is characteristic of mice with greater rotarod scores in A as compared to mice in (**B**). (**C**) Scatter plots for intra-session features with their best fit line.”

now reads:

“(**A**,**B**) Representative horizontal paw position changes over time for mice with greater (**A**) and smaller (**B**) rotarod scores. (**C**,**D**) Scatter plots for intra-session features with their best fit line.”

The original Article has been corrected.

